# Linking alterations in estrogen receptor expression to memory deficits and depressive behavior in an ovariectomy mouse model

**DOI:** 10.1038/s41598-024-57611-z

**Published:** 2024-03-21

**Authors:** Dong-Cheol Baek, Ji-Yun Kang, Jin-Seok Lee, Eun-Jung Lee, Chang-Gue Son

**Affiliations:** 1 Institute of Bioscience & Integrative Medicine, Daejeon Korean Hospital of Daejeon University, Daedukdae-ro 176 bun-gil 75, Daejeon, 35235 Republic of Korea; 2 Department of Korean Rehabilitation Medicine, Daejeon Korean Hospital of Daejeon University, Daedukdae-ro 176 bun-gil 75, Daejeon, 35235 Republic of Korea

**Keywords:** Neuroscience, Diseases, Endocrinology

## Abstract

The high risk of neurological disorders in postmenopausal women is an emerging medical issue. Based on the hypothesis of altered estrogen receptors (ERα and β) after the decline of estrogen production, we investigated the changes in ERs expressions across brain regions and depressive/amnesic behaviors. C57BL/6J female mice were ovariectomized (OVX) to establish a menopausal condition. Along with behavior tests (anxiety, depression, and memory), the expression of ERs, microglial activity, and neuronal activity was measured in six brain regions (hippocampus, prefrontal cortex, striatum, raphe nucleus, amygdala, and hypothalamus) from 4 to 12 weeks after OVX. Mice exhibited anxiety- and depressive-like behaviors, as well as memory impairment. These behavioral alterations have been linked to a suppression in the expression of ERβ. The decreased ERβ expression coincided with microglial-derived neuroinflammation, as indicated by notable activations of Ionized calcium-binding adapter molecule 1 and Interleukin-1beta. Additionally, the activity of brain-derived neurotrophic factor (BDNF), particularly in the hippocampus, decreased in a time-dependent manner from 4 to 12 weeks post-OVX. Our study provides evidence shedding light on the susceptibility to memory impairment and depression in women after menopause. This susceptibility is associated with the suppression of ERβ and alteration of ERα in six brain regions.

## Introduction

Menopause marks the permanent cessation of menstrual cycles, which is an inevitable event for women. As a result of the rapid decline in estrogen levels before and after menopause, approximately 70% of women usually begin to experience postmenopausal syndrome, including various physical and psychological problems^1^. Besides the rapid reductions of bone mineral density, in particular, memory dysfunction and significant mood changes frequently become medical issues for postmenopausal women^2^.

The association between estrogen deficiency and neuropsychiatric or neurodegenerative disorders has been well-recognized^3^. The studies overwhelmingly report that the incidence of Alzheimer’s disease (AD) does not differ by sex, while when women reach menopause, they are at a higher risk than men of developing AD^4^. Clinical studies have shown that estrogen has been found to promote neuroprotection and neural plasticity in AD patients, while low estrogen can be linked to memory loss^5,6^. Other clinical studies have shown that estrogen affects neurotransmitters such as serotonergic and noradrenergic circuitry, which play crucial roles in mood regulation^7,8^. The declined estrogen level of menopausal women presents a 2–5 fold higher risk of developing depression^4^.

Estrogen deficiency-derived neuroinflammation is proposed to alter both hippocampal plasticity and estrogen receptors (ERs), resulting in the exacerbation of depressive symptoms and memory loss^9,10^. Generally, estrogen actions rely on the binding to estrogen receptors alpha and/or beta (ERα and β) in various tissues including brain, contributing to different processes of cognition, mood, and sexual behavior^11^. From preclinical studies, ERα knockout mice showed severe deficits in reproduction^12^, while ERβ knockout mice presented memory loss and anxiety- and depressive-like behaviors^13^. In OVX rats, an ERβ agonist significantly attenuated depressive behavior compared to an ERα agonist^14^. In particular, ERβ is expressed at high levels at the regions of the hippocampus as a primary regulator of hippocampus functions in both rodents and humans^15,16^. Extensive neuroimaging studies reported that the hippocampus is the most frequently deficient region in patients with neuropsychiatric or neurodegenerative disorders^17^.

From the above facts, we could anticipate the strong link between depressive symptoms and memory loss after menopause and the alteration of ERs expression in the brain, however, no experimental information exists. Herein, the present study aimed to explore those questions using comprehensive and comparative analyses of ERs expression across multiple brain regions along with behavior tests in an ovariectomy mouse model.

## Results

### OVX induced anxiety and depressive-like behaviors

**Figure 1:**
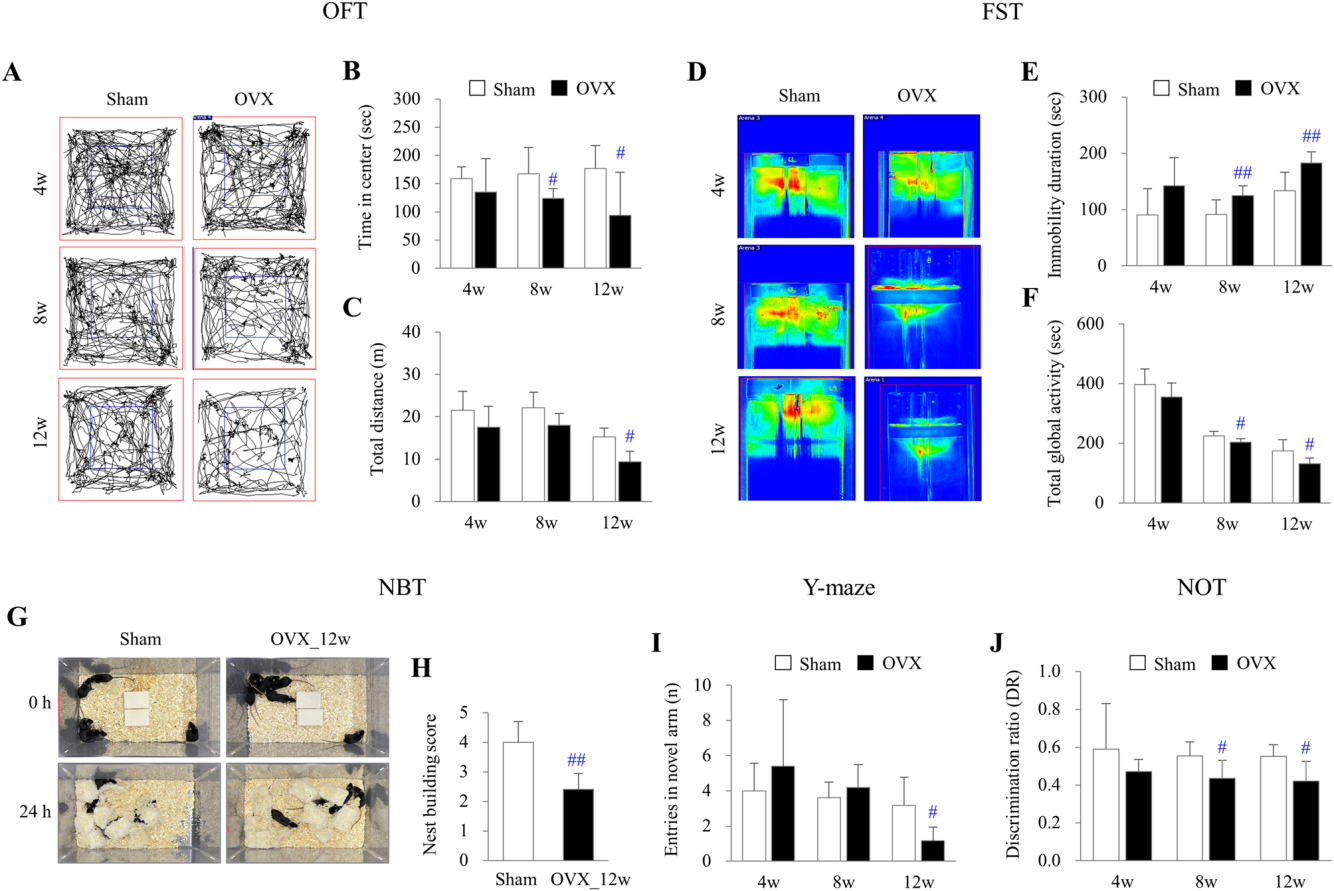
Depressive-like behaviors and memory impairment. From 4 to 12 weeks post-OVX, representative 2D tracking images (**A**), the time spent in the center (**B**), and the total distance (**C**) in the OFT, along with representative FST 2D tracking images (**D**), immobility duration (**E**), and total global activity (**F**) in the FST, and NBT images (**G**) and nest building score (**H**), were conducted to confirm that OVX induced anxiety and depressive-like behaviors in mice, respectively. In addition, the Y-maze test (**I**) and novel object test (**J**) were performed to confirm that OVX induced short- and long-term memory impairment in mice, respectively. The data were analyzed by unpaired t-test for the difference between sham and OVX at the same time-point (Each, n = 6). #p < 0.05 and ##p < 0.01 compared with the sham group.

Both behavior tests for depression (OFT) and anxiety (FST and NBT) evidenced that OVX presented anxiety and depressive-like behaviors at 12 weeks (p < 0.05 or p < 0.01). But not at 4 weeks and 8 weeks, compared with the sham group, respectively (Fig. 1A–H).

### OVX induced both short- and long-term memory impairment

After OVX, mice also showed a significant loss of memory function, for both short-term (Y maze test) and long-term memory (NOT) at 12 weeks (*p* < 0.05), but not at 4 weeks and 8 weeks, compared with sham group, respectively (Fig. 1I, J).

### OVX reduced the expression of ERβ in brain regions

**Figure 2:**
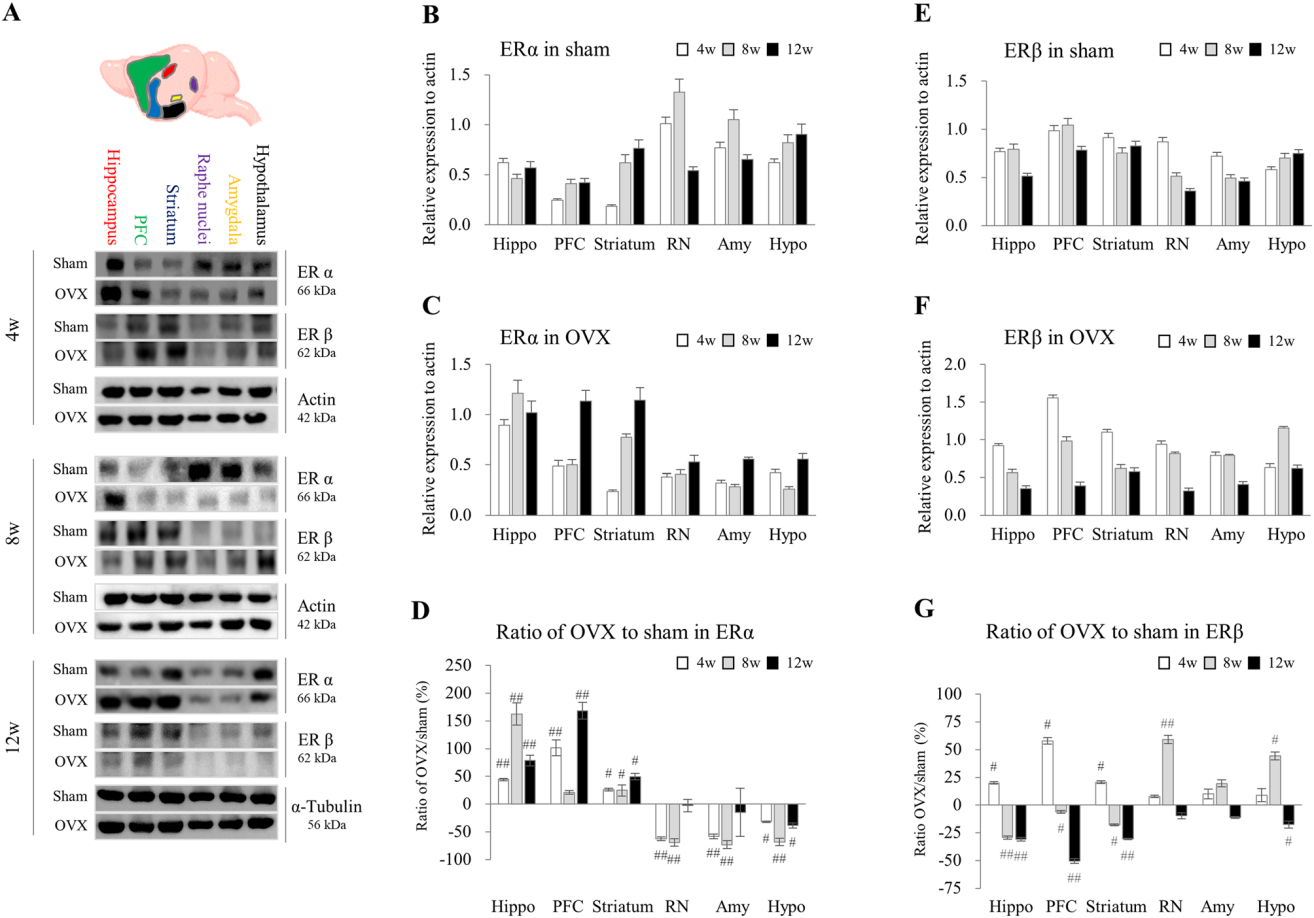
Changes in ER α and β in six brain regions. In OVX-induced and sham mice, the protein expression of ERα and β in the six brain regions were determined by western blotting from 4 to 12 weeks (**A**), and their intensities of ERα in sham group (**B**), ERα in OVX group (**C**), ratio of OVX to sham in ERα (**D**), ERβ in sham group (**E**), ERβ in OVX group (**F**), and ratio of OVX to sham in ERβ (**G**) were semi quantified using Image J. Hippo, hippocampus; PFC, prefrontal cortex; stri, striatum; RN, raphe nuclei; Amy, amygdala, and Hypo, hypothalamus. The data were analyzed by unpaired t-test for the difference between sham and OVX at the same time-point (Each, n = 6). #p < 0.05 and ##p < 0.01 compared with the sham group.

After 4–12 weeks of OVX, both protein expressions of ERα and ERβ were dynamically changed depending on each region of 6 brain regions. Compared with sham-operated mice, OVX increased ERα expression levels at the hippocampus, PFC, and striatum, but decreased at raphe nuclei, amygdala, and hypothalamus (Fig. 2A, B and E). On the contrary, ERβ expression was notably decreased in the hippocampus, PFC, and striatum as an augmented pattern by time pass (at 12 weeks), but not significant in other regions (Fig. 2A, C, D, F and G).

### OVX induced microglial-derived neuroinflammation in the brain regions

**Figure 3:**
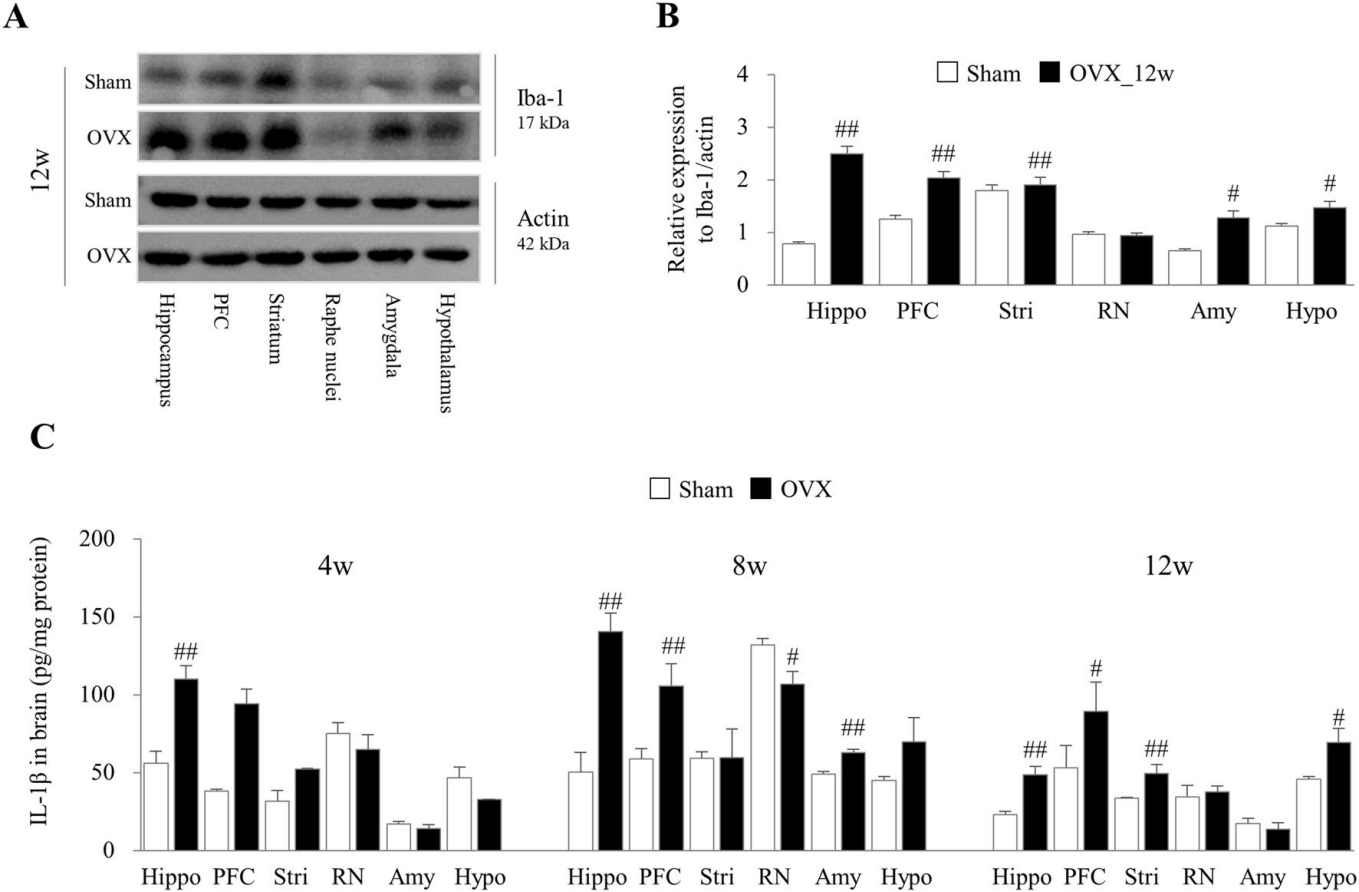
Alteration of microglial-derived neuroinflammation. In OVX-induced and sham mice, the protein expression of Iba-1 in the six brain regions was determined by western blotting (**A**), and their intensities of Iba-1 (**B**) at 12 weeks were semi-quantified using Image J, as well as the levels of IL-1β (**C**) in serum were analyzed ELISA from 4 to12 weeks. Hippo, hippocampus; PFC, prefrontal cortex, stri, striatum; RN, raphe nuclei; Amy, amygdala, and Hypo, hypothalamus. The data were analyzed by unpaired t-test for the difference between sham and OVX at the same time-point (Each, n = 6). #p < 0.05 and ##p < 0.01 compared with the sham group.

At 12 weeks after OVX, the notably activated microglia were observed at 5 brain regions (except raphe nuclei) compared to the sham group (*p* < 0.05 or *p* < 0.01), as evidenced by results from Iba-1 expression levels (Fig. 3A and B). In addition, IL-1β levels also significantly increased in various brain regions compared to the sham group from 4 to 12 weeks (*p* < 0.05 or *p* < 0.01, Fig. 3C).

### OVX altered hippocampal neuronal activity and levels of serotonin

**Figure 4:**
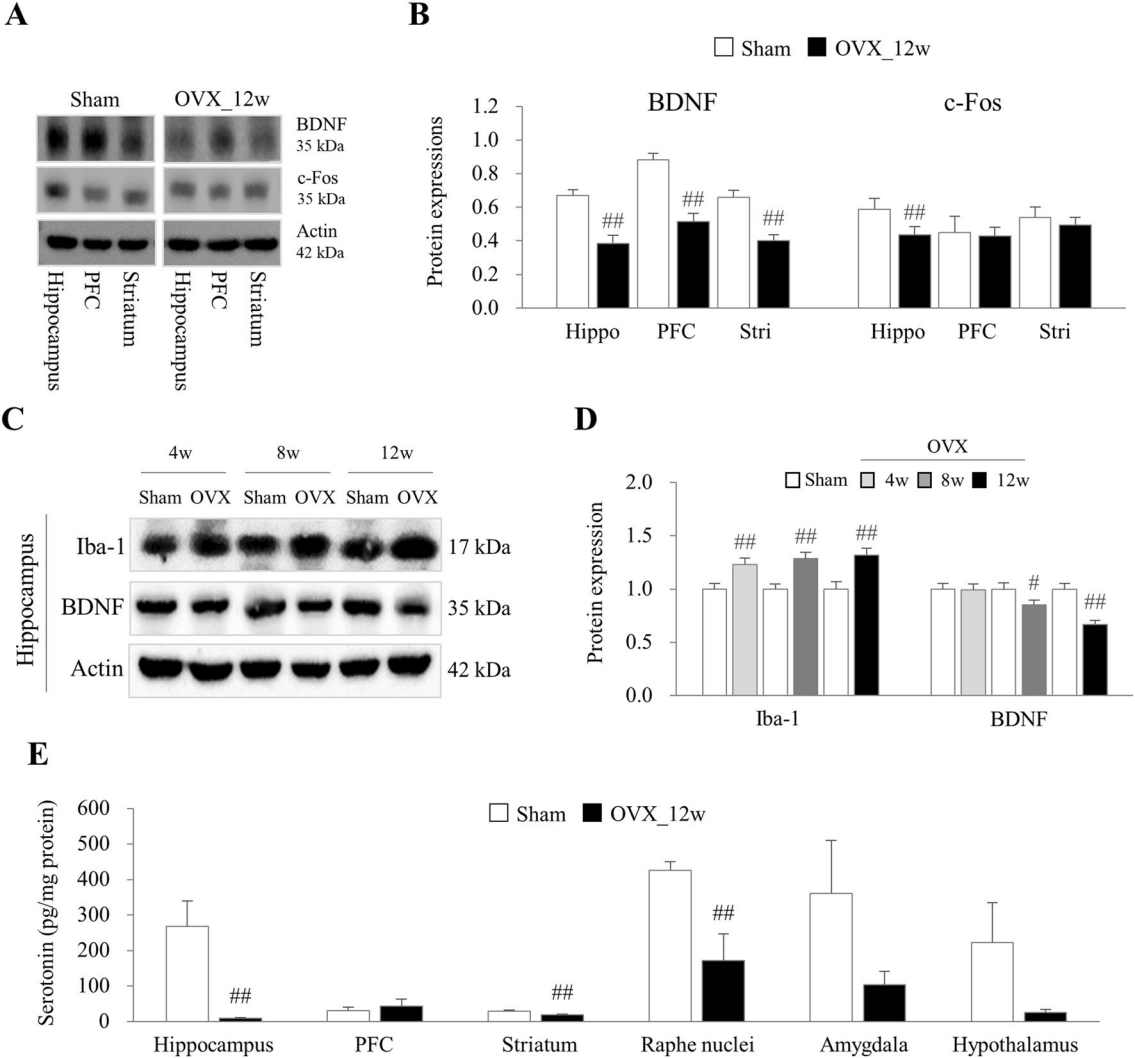
Alteration of neuronal activity and levels of serotonin. The protein expression of BDNF and c-Fos in the hippocampus, PFC, and striatum regions was assessed by western blot (**A**) and their intensities of BDNF and c-Fos (**B**) at 12 weeks as well as from 4 to 12 weeks, in the hippocampus, the protein expression of iba-1 and BDNF was assessed by western blot (**C**) and their intensities of iba-1 and BDNF (**D**) were semi-quantified using Image J. In addition, the levels of serotonin (**E**) in six brain regions were analyzed ELISA at 12 weeks. Hippo, hippocampus; PFC, prefrontal cortex; stri, striatum; RN, raphe nuclei; Amy, amygdala; and Hypo, hypothalamus. The data were analyzed by unpaired t-test for the difference between sham and OVX at the same time-point (Each, n = 6). #p < 0.05 and ##p < 0.01 compared with the sham group.

When compared to the neuronal activity-related protein expressions, OVX markedly suppressed the expression of BDNF especially in the hippocampus (*p* < 0.01) including PFC and striatum at 12 weeks (*p* < 0.01), along with only hippocampal c-Fos (*p* < 0.01, Fig. 4A and B). From the additional analyses, the decreased BDNF protein activity was decreased in a time-dependent manner after OVX (*p* < 0.05 or *p* < 0.01), while microglial activation (iba-1 over-expression) persisted from 4 to 12 weeks of OVX (*p* < 0.01, Fig. 4C and D). At 12 weeks, OVX significantly decreased serotonin levels in three brain regions (hippocampus, striatum, and raphe nuclei), with the hippocampus exhibiting the lowest serotonin levels (*p* < 0.05 or *p* < 0.01, Fig. 4E).

## Discussion

To obtain an experimental clue to explain the depressive symptoms and memory loss after estrogen-deficiency conditions, we conducted comprehensive and comparative analyses of ER expression across multiple brain regions using the OVX mouse model. The OVX model is well known to mimic estrogen deficiency-related clinical symptoms^18^. As expected, the OVX mice presented increased FSH in serum and body weight gain (Supplementary Fig. S1A and B), which are the laboratory and clinical features in estrogen-deficiency conditions^19^. Due to the lack of ovarian estradiol production and its inhibitory feedback, FSH levels may rise in an attempt to stimulate the ovaries^20^. We also identified the appearance of anxiety- and depressive-like behaviors as well as memory impairment after time passage of estrogen depletion (at 12 weeks of OVX), respectively (Fig. 1). Similar to our data, OVX mice showed a significant decrease in novel-object recognition and were immobile during the forced swim test, reflecting depression-like behaviors and memory impairment^21,22^. These behavioral and cognitive alterations are evidenced by suppression of serotonin levels in the serum as well as in three brain regions, most prominently the hippocampus (Fig. 4E and Supplementary Fig. S1C). The estrogen level has a significant impact on the emotional state and cognitive abilities of women throughout their lifespan, therefore, estrogen deficiency increases the vulnerability to mood and memory disorders in both humans and rodents^23,24^. These induction of depressive behavior and memory impairment in OVX-induced mice may be influenced by estrogen deficiency, alterations in neurotransmitter levels like serotonin, neuroinflammation, and the dysregulation of signaling pathways such as Nrf2/HO-1^25–27^.

Neuroinflammation is proposed as an important potential mechanism to explain the development of depressive mood and memory loss after estrogen deficiency^28,29^. Given the known fact that estrogen inhibits the inflammatory response^30^, menopausal women have elevated levels of proinflammatory cytokines such as TNF-α, IL-1β, and IL-8, increasing their risk of inflammatory disorders compared to fertile women.^31^. The presence of the inflammasome complex within the cerebrospinal fluid in postmenopausal women suggests a pro-inflammatory state in the brain^32^. As anticipated, mice at 12 weeks post-OVX exhibited overactivity of Iba-1 (an indicator of glial activation) and increased IL-1β, especially in three regions (hippocampus, PFC, and striatum) (Fig. 3A–C). Similar to our data, the activation of the NLRP3 inflammasome in the hippocampus has been associated with depression-like behavior and cognitive impairment in OVX mice^33,34^. In a recent clinical study, postmenopausal women exhibited neuroinflammation-related neuronal injury and disruption of neural circuits in the brain, which correlated with cognitive impairment and depression^35^.

Estrogenic actions depend on binding to ERα and ERβ in different brain regions, but their impact on expression remains controversial. the ERβ and its distribution of tissue and cellular expression are still unclear in various research fields^36^. In our data, both ERα and ERβ expression dynamically changed depending on each region of six brain regions in the sham mice from 4 to 12 weeks (Fig. 2A, B and E). Though the exact mechanism by which estrogen deficiency triggers neuroinflammation is not fully understood, recent studies have stressed the involvement of ERs in microglia and astrocytes^37,38^. ERα and ERβ are expressed in microglia and astrocytes, and then ERβ dominantly contributes to regulating neural generation, proliferation, and neuroinflammation^39^. An ERβ-selective agonist suppressed the production and release of pro-inflammatory cytokines by microglia and astrocytes in an in vitro study^40^. In middle-aged female mice (12–14 months), selective deletion of ERβ in astrocytes presented hippocampal neuroinflammation and cognitive impairment^41^. In the present study, mice 12 weeks post-OVX presented reduced ERβ expressions in all brain regions, most prominently in three regions (hippocampus, PFC, and striatum), but ERα expression remained unchanged (Fig. 2). These reductions in specific ERβ expression can significantly impair microglial functions, affecting their ability to maintain homeostasis and respond to infection and injury^37,42^. In ERβ knockout mice, the neuronal deficit becomes increasingly pronounced with age, leading to the degeneration of neuronal cell bodies throughout the brain by the age of 2 years^43^. Additionally, ERβ knockout mice showed increased anxiety-like behaviors in the elevated plus maze (EPM)^44,45^; however, OVX-induced rats treated with ERβ agonists showed reduced anxiety- and despair-like behaviors in the forced-swim test (FST)^46^.

To further investigate the impact of ERβ suppression on neuronal plasticity, we analyzed BDNF (an indicator of neuronal survival and growth) and c-Fos (an indicator of neuronal activity). Our findings revealed decreased expression of BDNF in three regions (hippocampus, PFC, and striatum) and a hippocampus-specific suppression of c-Fos (Fig. 4A and B). Both BDNF and c-Fos are crucial molecules involved in neuronal plasticity associated with learning and memory, and they have been proposed as potential markers influenced by psychiatric conditions^47,48^. Interestingly, our data suggest that estrogen deficiency has negative effects on neuronal survival and growth (BDNF) but is unaffected by neuronal activity (c-Fos). However, the observed reductions in both BDNF and c-Fos expression in the hippocampus suggest that neuronal growth and activity are particularly vulnerable to estrogen deficiency (Fig. 4A and B). In ERβ knockout mice, there were decreased activities of BDNF expression (40%) and significantly elevated 5-HT_2A_ receptor signaling in the hippocampal region^49^. Studies have reported an increase in 5-HT_2A_ receptor signaling in the hippocampus and prefrontal cortex of depressed patients^50,51^. In fact, the hippocampus has been well known as a pivotal region not only for learning and memory but also for mood regulation, including the serotonin circuit^52,53^. We confirmed the time-dependent decrease in BDNF expression but the overactivation of microglial in the hippocampus from 4 to 12 weeks of post-OVX (Fig. 4C and D). In addition, we also observed a decreased serotonin level in serum at 12 weeks, with the hippocampus exhibiting the most pronounced suppression (Fig. 4E and Supplementary Fig. S1C).

We herein provided the comparative analysis of ERα and ERβ expressions across six major brain regions and found suppressed ERβ, especially in the hippocampus, along with behavior tests of memory loss and depression. However, parallel to the limitations of the present study, the ERβ antibodies are still insufficiently validated in various research fields^36^. Furthermore, we need to conduct further experiments for direct evidence of the behavioral and glial activation effects of OVX in middle-aged mice, whether those altered behaviors can be prevented by restoring ERβ expression, and how ERβ suppression induces neuroinflammation in the future.

In conclusion, as our knowledge, we first may provide experimental clues through the OVX-induced mice to explain the memory impairment and depressive-like behavior of post-menopausal women, which are associated with the alteration of estrogen receptors.

## Materials and methods

### Chemicals and reagents

The reagents and chemicals were purchased from Sigma-Aldrich (St. Louis, MO, USA); Neutral formalin (10%), acetic acid, a bicinchoninic acid (BCA) protein assay kit, *N*-(1-naphthyl)-ethylenediamine dihydrochloride, sodium chloride, tetraethyl ethylene-diamine (TEMED), Trizma base, Triton X, and Tween 20.

Additional reagents and chemicals were obtained from the following manufacturers: 10% ammonium per-sulfate solution, radioimmunoprecipitation assay (RIPA) buffer, and skim milk were obtained from LPS Solution (Daejeon, Republic of Korea); bovine serum albumin (BSA) was obtained from GenDEPOT (Barker, TX, USA); 4% paraformaldehyde (PFA), 10X Tris–glycine buffer, and 10X Tris glycine-SDS buffer were obtained from XOGENE (Daejeon, Republic of Korea); protease inhibitor, phosphatase inhibitor, RNA Later, chemiluminescence (ECL) advanced kit and estrogen receptor alpha, estrogen receptor beta, and actin antibody were obtained from Thermo Fisher Scientific (Waltham, MA, USA); methylene alcohol was obtained from Daejung Chemicals & Metals Co. (Siheung, Republic of Korea); polyvinylidene fluoride (PVDF) membranes were obtained from Pall Co. (Port Washington, NY, USA).

### Animals and ovariectomy

A total of thirty-six female C57BL/6 J mice (6 weeks old, 15–17 g) were obtained from Dae Han Bio Link (Eumseong, Chungcheongbuk-do, Republic of Korea). All animals had ad libitum access to a commercial pellet diet (Cargill Agri Purina, Seongnam, Korea) and tap water and were housed at room temperature (22 ± 2 °C) and 60 ± 5% relative humidity under a 12 h light:12 h dark cycle. The study was approved by the guidelines of the Institutional Animal Care and Use Daejeon University Ethics Committee (Daejeon, Republic of Korea; Approval No. DJUARB2023-012) and carried out in strict accordance with the ARRIVE guidelines 2.0^54^.

The mice were used for experiments after acclimation for 7 days. Ovariectomy (OVX) surgery and experimental design were performed as follows^55^: Briefly, OVX-induced mice were intraperitoneally injected with a ketamine and xylazine mixture (90 mg/kg), had shaved skin, and were incised longitudinally to remove the bilateral ovaries, then the exposed skin and muscles were closed. Along with this, the sham mice were anesthetized in the same manner as the OVX group. They had shaved skin and were incised, and then the ovaries were exposed (no removal of ovaries). Finally, the surgical area was closed and disinfected with povidone-iodine.

After 7 days of postoperative recovery, the mice were divided into two groups (n = 6 for each group): Sham and OVX groups. The mice were euthanized using CO_2_ at four-week intervals (after OVX 4, 8, and 12 weeks) and the brain and serum were obtained. All tissues were stored at − 80 °C until use.

### Open field test (OFT)

Depressive and anxiety-like behavior was evaluated using an open-field test as previously described^56^. Before we tested, all animals were adapted to the dark-circadian phase for 2 h with 50-lx lighting conditions. We determined that mice exhibited depressive and anxiety-like behavior when they spent more time in the peripheral zone than in the center zone. Subsequently, their total distance and latency time in a central zone were recorded for 8 min at 50 lx using a video camera connected to the corresponding software. (Smart Junior).

### Forced swimming test (FST)

To determine depressive and anxiety-like behavior, a forced swimming test (FST) was examined. Before we tested, all animals were adapted to the dark-circadian phase for 2 h with 50-lx lighting conditions. The FST was performed using a cylindrical container 20 cm in diameter by 30 cm in height that was filled with water (24 ± 1 °C) to a depth of 20 cm. The mice were allowed to swim for 6 min in their containers. When a mouse initially floated upright after 2 min, making only small movements to maintain its head above water, latency time was measured. For 4 min, the number of times a mouse adopted an immobile position was recorded. We determined that the mice exhibited depressive-like behavior when their activity lasted less than 60 s^57^.

### Nest building test (NBT)

The investigation of anxiety-like behavior was conducted using the nested building test (NBT), as previously described^58^. In summary, ten squares of pressed cotton measuring 7 × 5 cm each were positioned in the center of the floor of a cage house, totaling 24 g. On a scale of 0–5, the degree to which the mice bit the squares, moved them into the corners and nested with the squares overnight was measured. The nest score of less than 2 was considered as a sickness condition, as described previously^59^.

### Novel object test (NOT)

Recognition memory was evaluated through a novel object test. The novel object test was adapted from the procedure described by Marianne Leger^60^. Before we train and test session, all animals were adapted to the dark-circadian phase for 2 h with 50-lx lighting conditions, and before 24 h of the training session, the mice were adapted to the open field (40 × 40 × 30 cm) for 10 min. In a training session, each mouse was allowed for 10 min with exposure to the same objects (2 × 4 × 10 cm) placed in the left and right corners of the arena. After the training session, the mice returned to their home cages and had a 6-h retention interval for rest and sleep. Each mouse was put into the field for the testing session, where one familiar object was replaced with a novel object (2 × 4 × 10 cm). The test session was recorded as a video, and the mouse was assigned 10 min of exploring. We recorded the time spent exploring each object on each mouse in the video. Exploration was defined as the animal’s nose is within 2 cm of and pointed toward the object. The time during which the animal propped itself upon the object to explore was not considered exploration time for that object. The discrimination ratio was calculated as the time spent with the novel object divided by the time spent exploring either object.

### Y maze test

Short-term spatial memory was assessed using a Y maze test. The Y maze test was performed as previously described with slight modification^61^. The Y maze was made of acrylic and consisted of three arms (60 × 60 × 15 cm) separated by 120°. Before we tested, all animals were adapted to the dark-circadian phase for 2 h with 50-lx lighting conditions. The process consists of a 2-min encoding trial that blocks one arm, a 1-min interval, and a 2-min test session. The starting arm remained the same for both encoding and test, the arm exposed during the encoding trial was considered a familiar arm, and the blocked arm was considered a novel arm. If the entire body crossed the entrance line, the mouse was considered to be on the arm. At the end of the test session. The short-term spatial memory was scored as the latency time to explore the novel arm in the Y maze.

### Western blot analysis

Western blot was performed to evaluate the expression of estrogen receptors in various tissues. The brains were homogenized with RIPA buffer. Prepared protein samples were separated by 10% polyacrylamide gel electrophoresis and transferred to polyvinylidene fluoride (PVDF) membranes using a Mini-PROTEAN Tetra Cell System (Bio-Rad, Hercules, CA, USA). After blocking in 5% skim milk at room temperature for 1 h, the membranes were incubated with primary antibodies against estrogen receptor-alpha (1:1000, MA1-310), estrogen receptor-beta (1:1000, PA1-311), actin (1:1000, MA5-116869), α-Tubulin (1:1000, ab7291), Ionized calcium-binding adapter molecule1(Iba-1, 1:1000, 016-20001) brain-derived neurotrophic factor (BDNF, 1:1000, ab108319) and c-Fos (1:1000, SC166940) at 4 °C overnight in a shaking plate. After washing with 0.1% TBS-T, the membranes were incubated with HRP-conjugated anti-mouse (1:5000, to detect estrogen receptor-alpha, actin, α-Tubulin and BDNF) or anti-rabbit (1:5000, to detect estrogen receptor-beta, Iba-1, and c-Fos) antibodies for 50 min. The membrane was then developed using an enhanced chemiluminescence (ECL) advanced kit (Thermo Fisher Scientific, Cleveland, OH, USA), and imaging was performed using a FUSION Solo System (Vilber Lourmat, France). Protein expression analysis was semi-quantified using Image J (NIH, MD).

### Enzyme-linked immunosorbent assay (ELISA)

After sacrificing, serum was immediately collected from the blood by centrifugation at 3000 rpm for 15 min at 4 °C and stored at − 80 °C until use. The Follicle-stimulating hormone (FSH) (E-EL-M0511, Elabscience, Houston, TX, USA) in serum, levels of interleukin 1-beta (IL-1β) (DY-401, R&D Systems, Minneapolis, MN, USA) and serotonin (BA E-5900, LDN, Germany) in brain regions (hippocampus, prefrontal cortex, striatum, raphe nucleus, amygdala, and hypothalamus) were measured using an enzyme-linked immunosorbent assay (ELISA) kit. The procedures were conducted according to the manufacturer’s instructions.

### Statistical analysis

The results were expressed as the mean ± standard deviation (SD) or fold changes in the means. In all analyses performed using GraphPad Prism 7 (GraphPad Software 9.4.1 version for Windows, San Diego, CA, USA, www.graphpad.com), p < 0.05 was considered to indicate statistical significance. All behavior tests (OFT, FST, NBT, Y-maze, and NOT, Fig. 1) and all western blot analyses (Figs. 3 and 4) were analyzed by unpaired t-test for the difference between sham and OVX at the same time-point.

In addition, the statistical analysis of estrogen receptor expression mean values in each brain region for sham (Fig. 2B, ERα and 2E, ERβ) and OVX (Fig. 2C, ERα and 2F, ERβ) was analyzed by an unpaired t-test at the same time point and then presented an amount of alteration in OVX to sham ratios (%) for ERα and ERβ. (Fig. 2D and G). Also, the calculation of the standard deviation for the ERα and ERβ ratio of OVX mice to sham mice was performed in the following manner (Fig. 2D and G)

$$ SD_{E,change} = \sqrt {SD_{E,baseline}^{2} + SD_{E,final}^{2} - \left( {2{ } \times {\text{ Corr }} \times SD_{E,baseline} \times SD_{E,final} } \right)} $$

### Ethics declarations

The study was approved by the guidelines of the Institutional Animal Care and Use Daejeon University Ethics Committee (Daejeon, Republic of Korea; Approval No. DJUARB2023-012).

### Supplementary Information

Supplementary Figure 1.

Supplementary Figures.

## Data Availability

The original contributions presented in the study are included in the article/Supplementary Material, further inquiries can be directed to the corresponding authors.
