# Supplementary material for: Linking alterations in estrogen receptor expression to memory deficits and depressive behavior in an ovariectomy mouse model
Source: Sci Rep. 2024 Mar 21;14:6854. doi: 10.1038/s41598-024-57611-z (PMC10958029; doi:10.1038/s41598-024-57611-z)
Supplement: Supplementary file 1 — Supplementary Figure 1. [file 41598_2024_57611_MOESM1_ESM.pptx]

## Slide 1
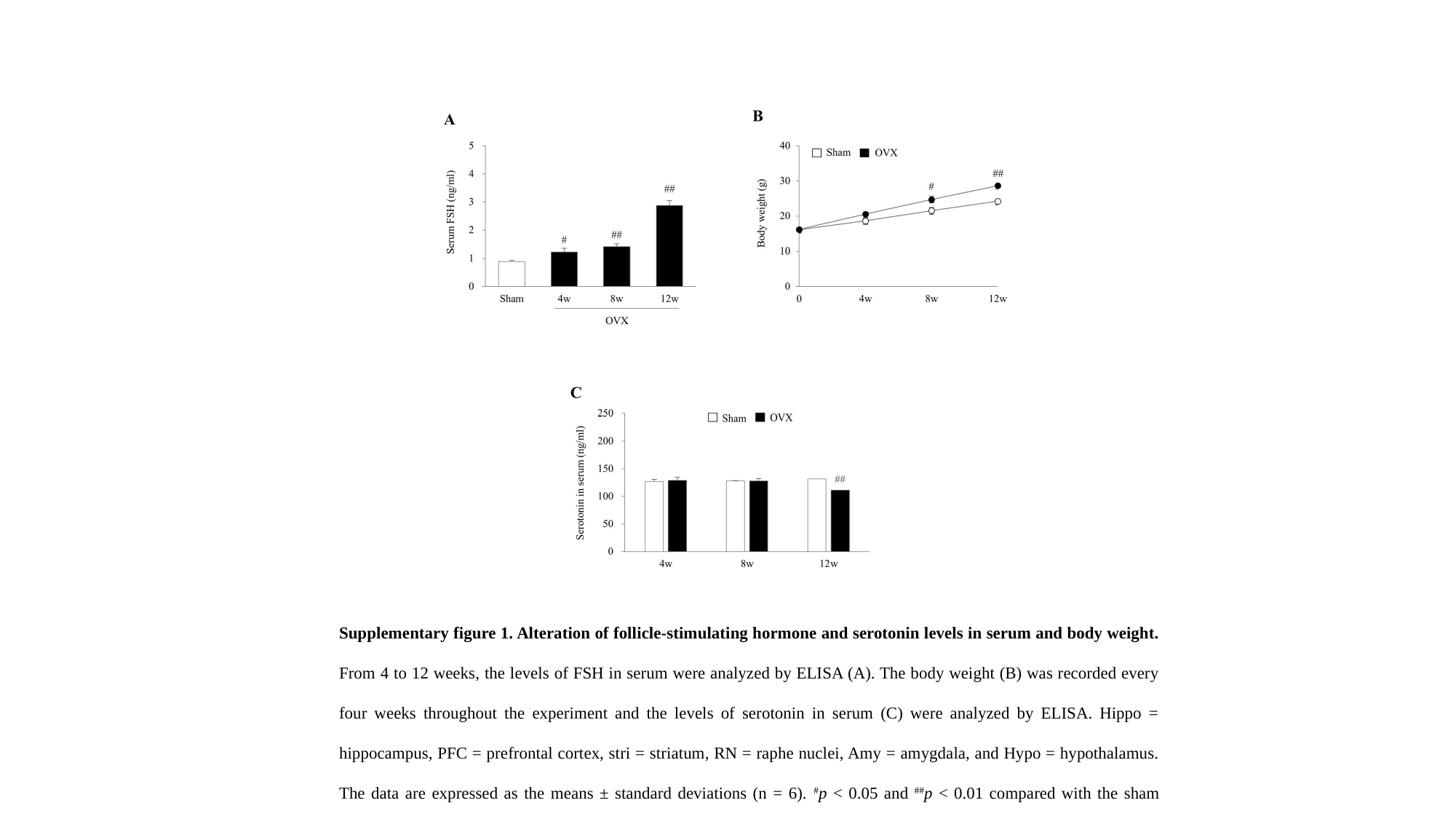

Supplementary figure 1. Alteration of follicle-stimulating hormone and serotonin levels in serum and body weight. From 4 to 12 weeks, the levels of FSH in serum were analyzed by ELISA (A). The body weight (B) was recorded every four weeks throughout the experiment and the levels of serotonin in serum (C) were analyzed by ELISA. Hippo = hippocampus, PFC = prefrontal cortex, stri = striatum, RN = raphe nuclei, Amy = amygdala, and Hypo = hypothalamus. The data are expressed as the means ± standard deviations (n = 6). #p < 0.05 and ##p < 0.01 compared with the sham group.
