# Supplementary figures and images for: Linking alterations in estrogen receptor expression to memory deficits and depressive behavior in an ovariectomy mouse model
Source: Sci Rep. 2024 Mar 21;14:6854. doi: 10.1038/s41598-024-57611-z (PMC10958029; doi:10.1038/s41598-024-57611-z)

**Original images for western blot**


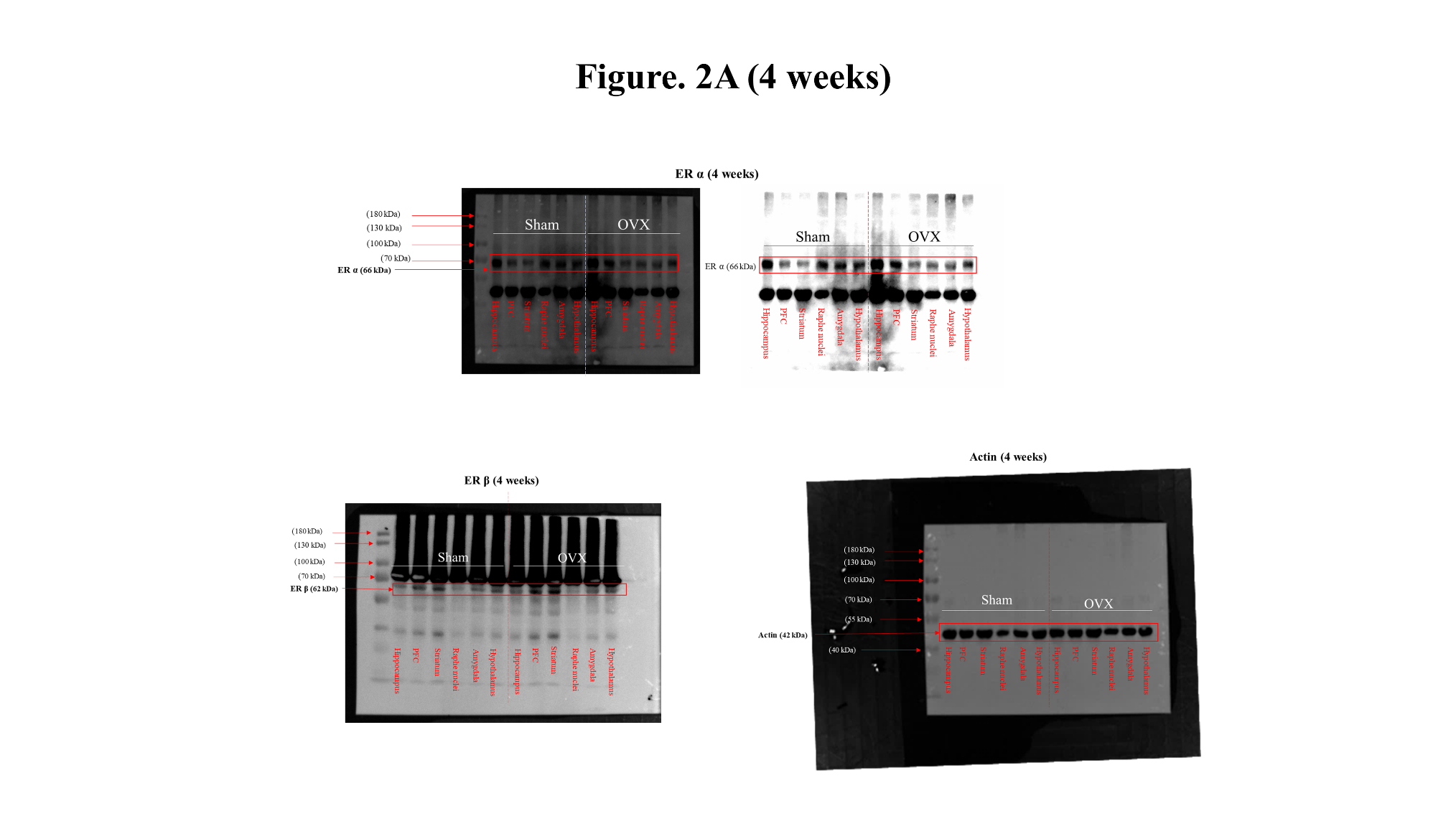


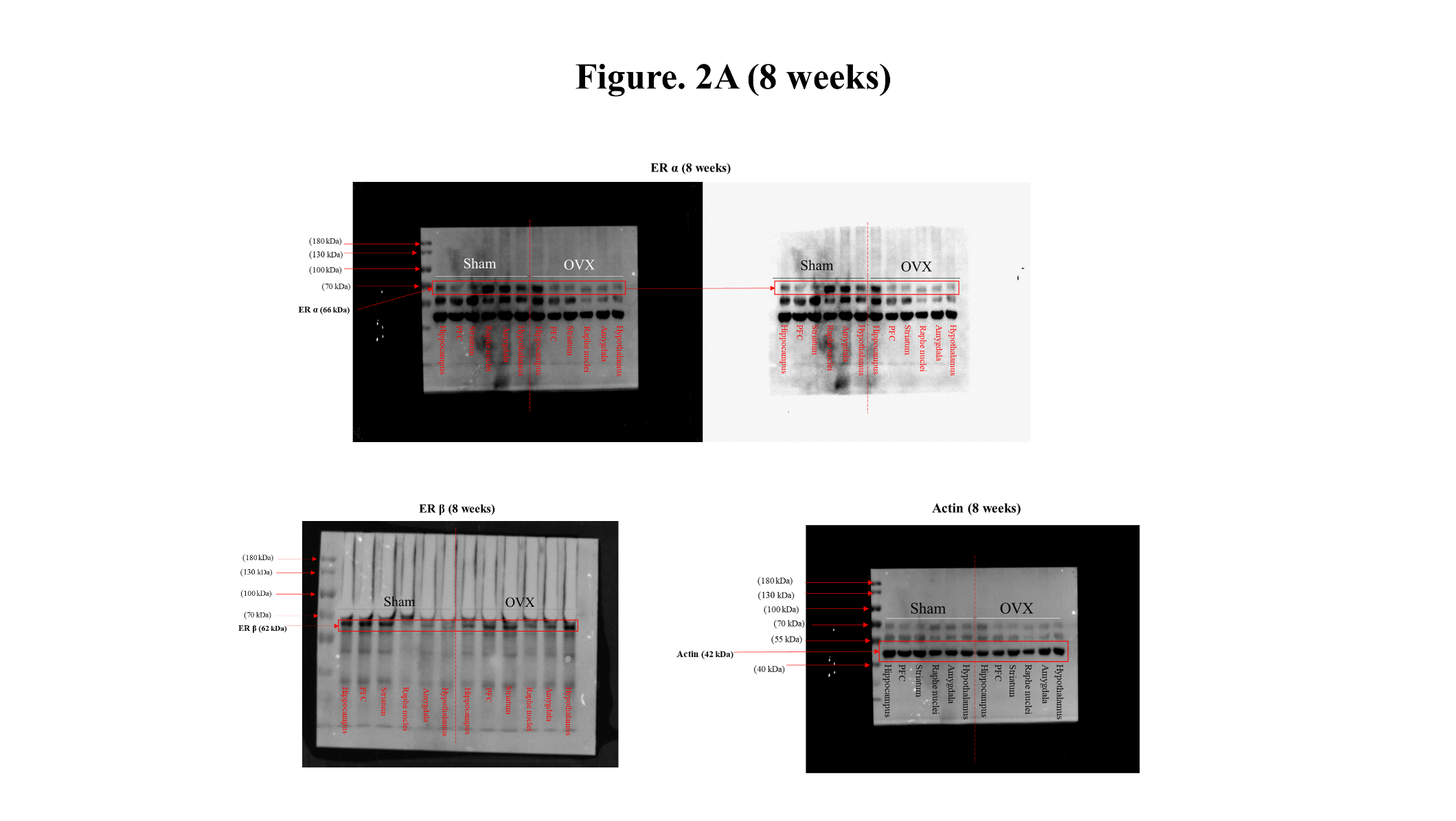


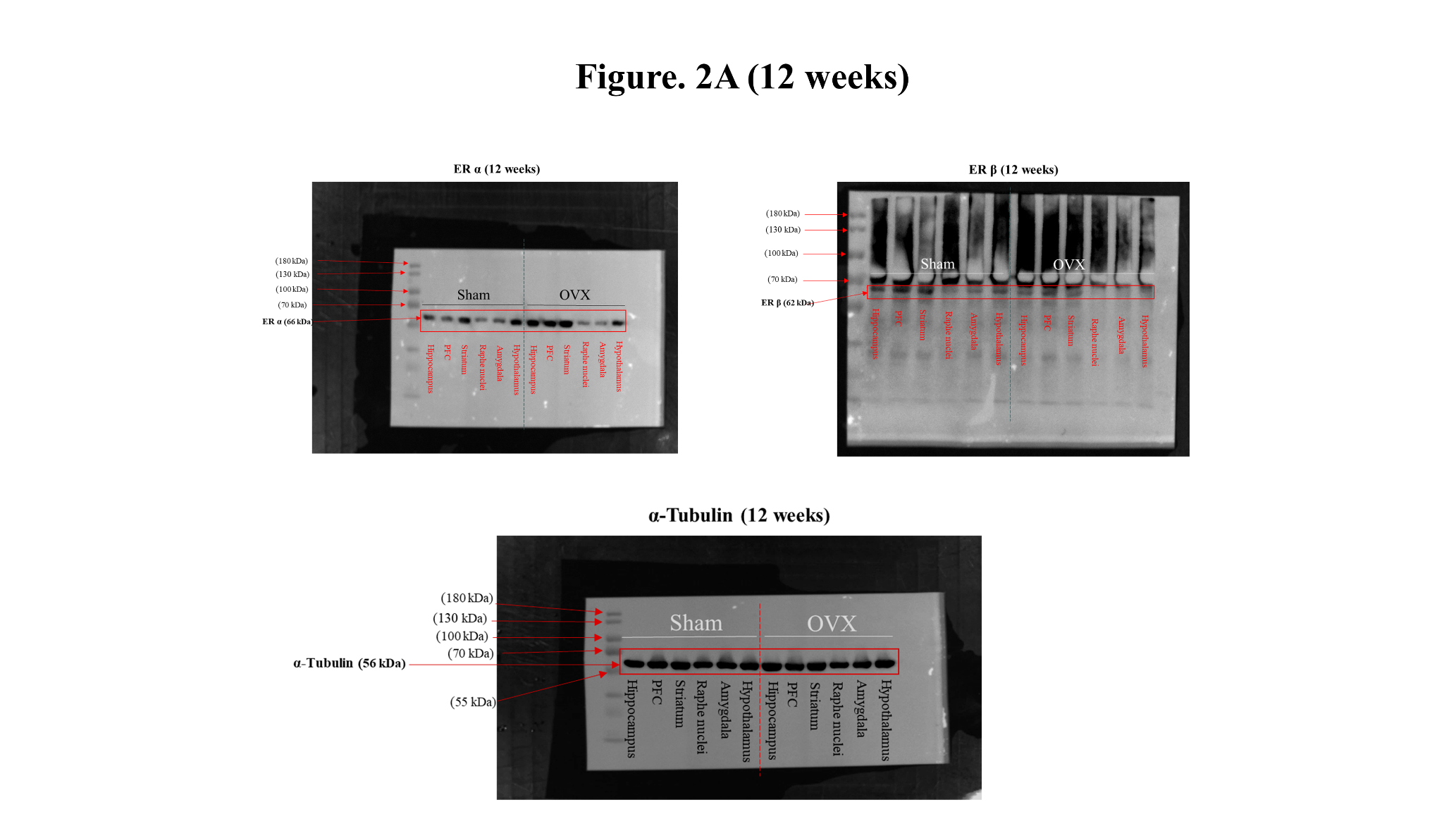


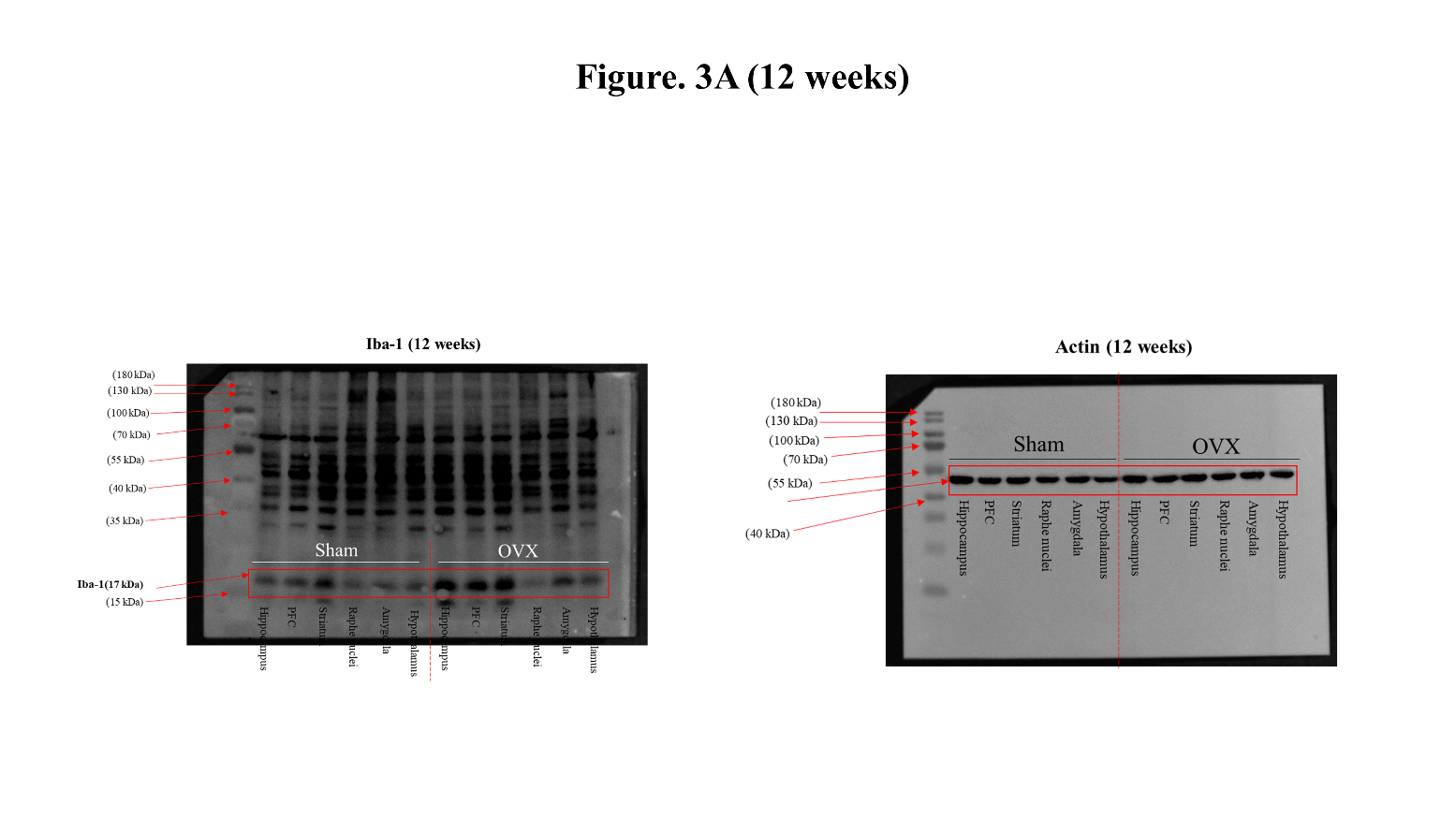


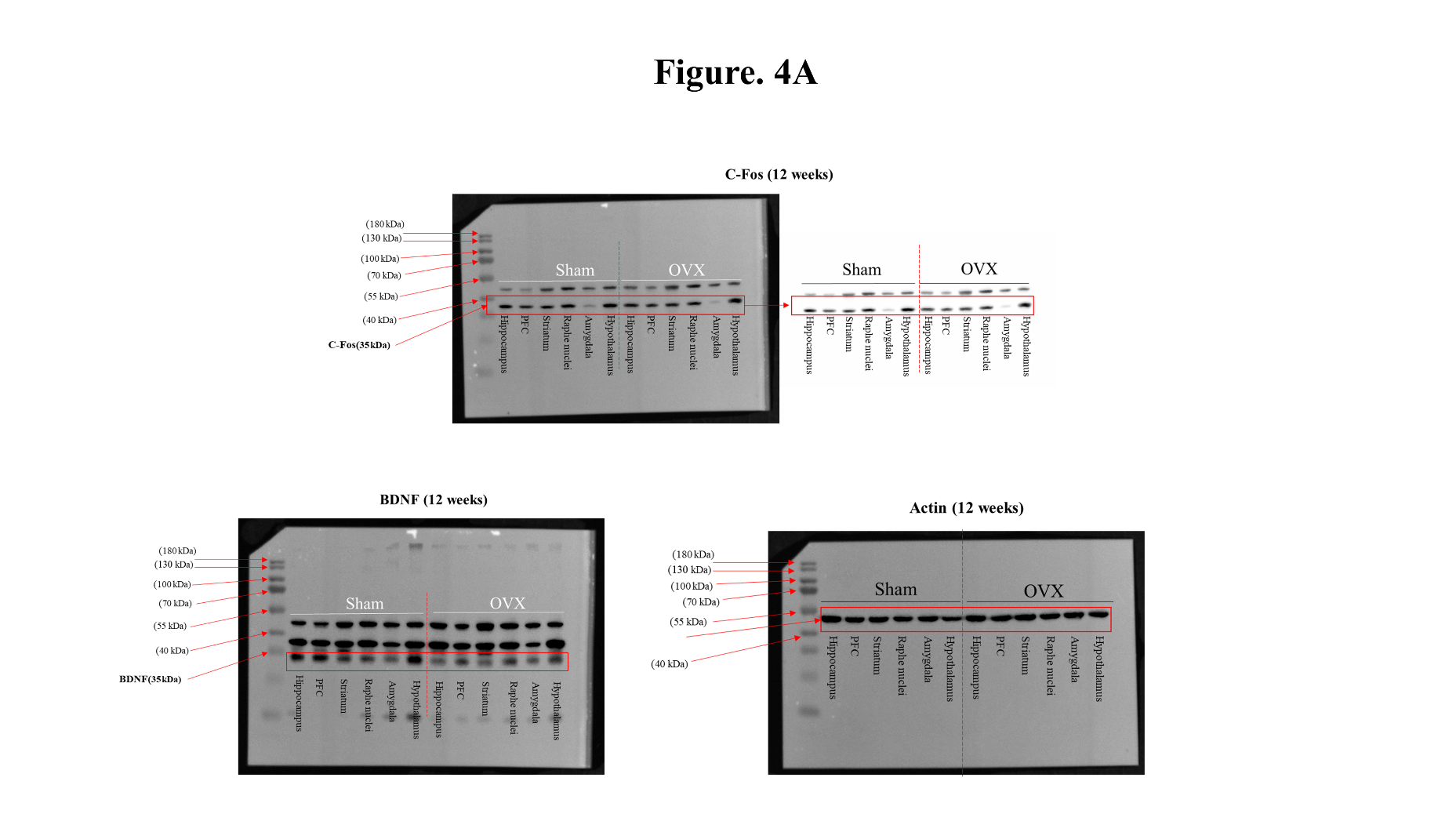


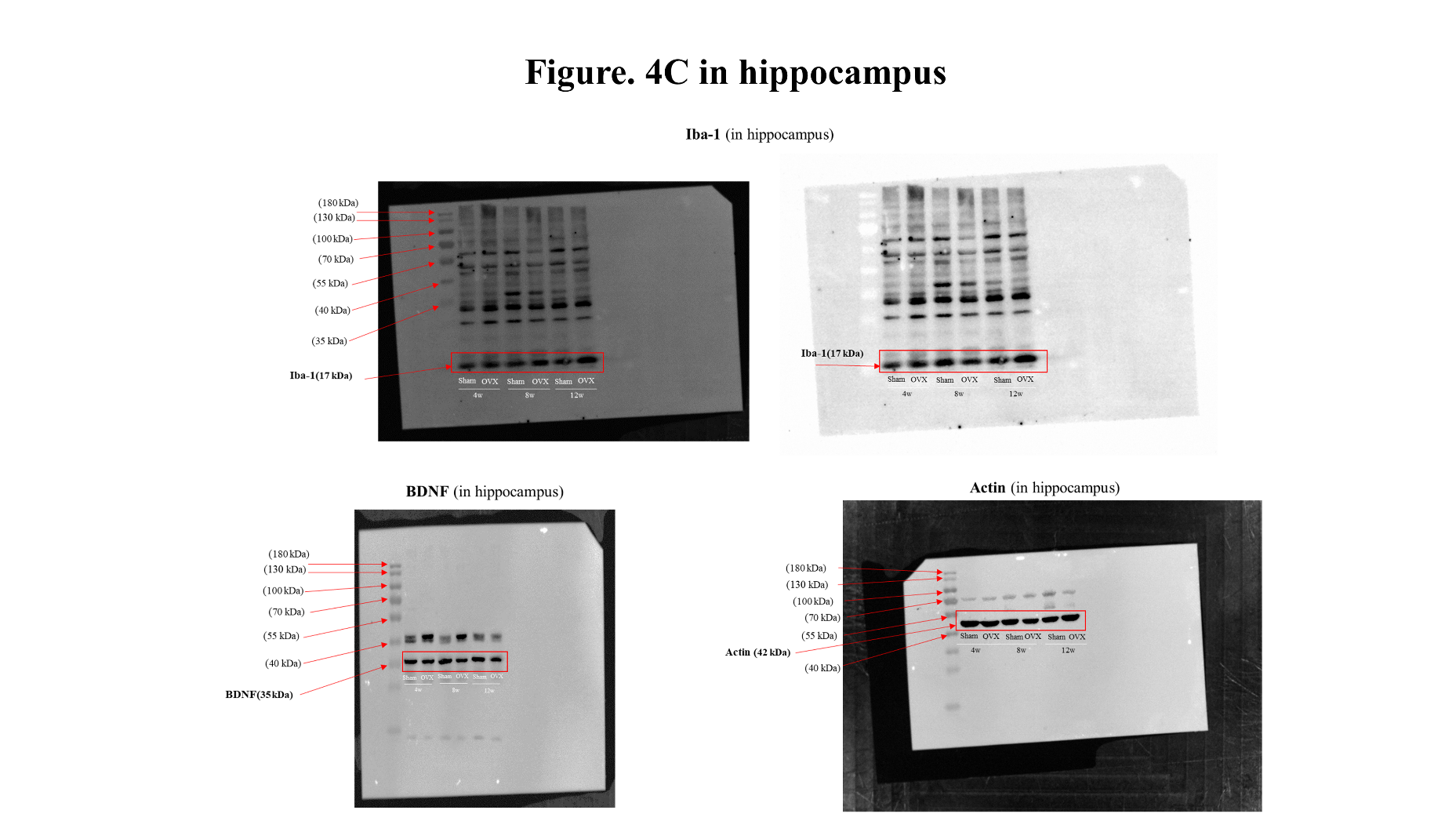

Supplement: Supplementary file 2 — Supplementary Figures. [file 41598_2024_57611_MOESM2_ESM.docx]
